# Supplementary material for: Defining host–pathogen interactions employing an artificial intelligence workflow
Source: eLife. 2019 Feb 12;8:e40560. doi: 10.7554/eLife.40560 (PMC6372283; doi:10.7554/eLife.40560)
Supplement: Supplementary file 1. [file elife-40560-supp1.docx]

**Supplementary File 1:**

**Table S1: Overview and evaluation of existing software packages for analysis of fluorescence images in HCI experiments.**

| **Program** | **Reference** | **Machine learning and deep learning features and user friendliness** |
| --- | --- | --- |
| CellProfiler Analyst (CA) | Carpenter *et al*, 2006 | Deep learning (DL) is not a native part of CA. Using it requires writing python code. Furthermore, due to necessity to maintain the integration between DL framework (e.g. Google TensorFlow) and CA such code is even more complex defeating the purpose of the framework for DL application. |
| WND-CHARM | Orlov *et al*, 2008 | This collection of scripts requires coding and integration with up-to date libraries. |
| CellClassifier | Rämö *et al*, 2009 | Being a milestone research contribution, CellClassifier is a discontinued legacy solution, making it currently unusable. |
| CellCognition (CC) | Held *et al*, 2010 | CC is a SVM-based user-friendly machine learning classification tool. However, similar to CA, DL is not a native part of CC, requires coding and comes with compatibility overhead. |
| Enhanced Cell Classifier (ECC) | Misselwitz *et al*, 2010 | While employing machine Learning (ML), ECC uses simplistic Support Vector Machine (SVM) classifier, which is not on par with modern machine learning techniques. ECC is a legacy solution. |
| Ilastik | Sommer *et al*, 2011 | Ilastik is ML-first oriented image analysis software with a great training interface and graphic processing unit (GPU) acceleration. It provides use of Random Forest ML classifier and SVM but has no DL support. |
| PhenoRipper | Rajaram *et al*, 2012 | PhenoRipper provides a lightweight analytical alternative to conventional computer vision approaches. However, it does not include the latest methodology like DL. |
| cellXpress (CX) | Laksameethanasan *et al*, 2013 | CX offers great performance based on conventional computer vision algorithms. It is equipped with principle component analysis and SVM-based machine learning, however has no DL integration available. |
| Cytomine | Marée & et al., 2013 | Cytomine is a great implementation of python basic ML libraries. However, it is based on the Scikit-learn library, which is missing DL. Furthermore, it is cloud only, which is impractical for large-scale HCS datasets and/ or unpublished data. |
| PhenoDissim | Zhang & Boutros, 2013 | PhenoDissim is a tool to analyze phenotypic dissimilarity based on SVM, this approach has not been followed up with more advance ML techniques like DL. |
| BioConductor (BC) | Huber *et al*, 2015 | BC is an important tool in Bioinformatics with a substantial bioimage informatics capabilities. However, using BC requires coding. Furthermore, being aimed for R rather than python it provides poor integration with modern day DL libraries. |
| CP-CHARM | Uhlmann *et al*, 2016 | Elaborated pure code analysis solution. However, requires understanding the code and integration with modern day libraries. |
| Advanced Cell Classifier (ACC) | Piccinini *et al*, 2017 | ML in ACC solution is based on a simplistic multi-layer perceptron network (MLP), which has been shown to be incapable of high complexity learning. |
| HTX | Arteta *et al*, 2017 | Great tool combining DL and graphical user interface in Matlab, however it lacks specific host-pathogen analysis. Furthermore, Matlab usage requires additional commercial licenses for highly specific toolboxes used here, which significantly limits applicability of this framework. |
| Trainable Weka Segmentation | Arganda-Carreras *et al*, 2017 | Weka has the largest ML classifiers collection, however for image analysis it relies on either ImageJ or KNIME integration and in absence of GPU acceleration no DL integration. |

**References**

Arganda-Carreras I, Kaynig V, Rueden C, Eliceiri KW, Schindelin J, Cardona A & Sebastian Seung H (2017) Trainable Weka Segmentation: a machine learning tool for microscopy pixel classification. *Bioinformatics* **33:** 2424–2426

Arteta C, Lempitsky V, Zak J, Lu X, Noble A & Zisserman A (2017) HTX: a tool for the exploration and visualization of high-throughput image assays. *bioRxiv***:** 204016

Carpenter AE, Jones TR, Lamprecht MR, Clarke C, Kang IH, Friman O, Guertin DA, Chang JH, Lindquist RA, Moffat J, Golland P & Sabatini DM (2006) CellProfiler: image analysis software for identifying and quantifying cell phenotypes. *Genome Biol.* **7:** R100

Held M, Schmitz MHA, Fischer B, Walter T, Neumann B, Olma MH, Peter M, Ellenberg J & Gerlich DW (2010) CellCognition: time-resolved phenotype annotation in high-throughput live cell imaging. *Nat. Methods* **7:** 747–754

Huber W, Carey VJ, Gentleman R, Anders S, Carlson M, Carvalho BS, Bravo HC, Davis S, Gatto L, Girke T, Gottardo R, Hahne F, Hansen KD, Irizarry RA, Lawrence M, Love MI, MacDonald J, Obenchain V, Oleś AK, Pagès H, et al (2015) Orchestrating high-throughput genomic analysis with Bioconductor. *Nat. Methods* **12:** 115–21

Laksameethanasan D, Tan R, Toh G & Loo L-H (2013) cellXpress: a fast and user-friendly software platform for profiling cellular phenotypes. *BMC Bioinformatics* **14:** 1–12

Marée R & et al. (2013) Extremely randomized trees and random subwindows for image classification, annotation, and retrieval. and Pat. *Decis. For. Comput. Vis. Med. Image Anal. Adv. Comput. Vis. Pattern Recognit.***:** 125–142

Misselwitz B, Strittmatter G, Periaswamy B, Schlumberger MC, Rout S, Horvath P, Kozak K & Hardt W-D (2010) Enhanced CellClassifier: a multi-class classification tool for microscopy images. *BMC Bioinformatics* **11:** 30

Orlov N, Shamir L, Macura T, Johnston J, Eckley DM & Goldberg IG (2008) WND-CHARM: Multi-purpose image classification using compound image transforms. *Pattern Recognit. Lett.* **29:** 1684–1693

Piccinini F, Balassa T, Szkalisity A, Molnar C, Paavolainen L, Kujala K, Buzas K, Sarazova M, Pietiainen V, Kutay U, Smith K & Horvath P (2017) Advanced Cell Classifier: User-Friendly Machine-Learning-Based Software for Discovering Phenotypes in High-Content Imaging Data. *Cell Syst.* **4:** 651–655.e5

Rajaram S, Pavie B, Wu LF & Altschuler SJ (2012) PhenoRipper: software for rapidly profiling microscopy images. *Nat. Methods* **9:** 635–637

Rämö P, Sacher R, Snijder B, Begemann B & Pelkmans L (2009) CellClassifier: supervised learning of cellular phenotypes. *Bioinforma. Appl. NOTE* **25:** 3028–303010

Sommer C, Straehle C, Kothe U & Hamprecht FA (2011) Ilastik: Interactive learning and segmentation toolkit. In *2011 IEEE International Symposium on Biomedical Imaging: From Nano to Macro* pp 230–233. IEEE

Uhlmann V, Singh S & Carpenter AE (2016) CP-CHARM: segmentation-free image classification made accessible. *BMC Bioinformatics* **17:** 51

Zhang X & Boutros M (2013) A novel phenotypic dissimilarity method for image-based high-throughput screens. *BMC Bioinformatics* **14:** 336
